# Chronic Sleep Restriction While Minimizing Circadian Disruption Does Not Adversely Affect Glucose Tolerance

**DOI:** 10.3389/fphys.2021.764737

**Published:** 2021-10-20

**Authors:** Robin K. Yuan, Kirsi-Marja Zitting, Jeanne F. Duffy, Nina Vujovic, Wei Wang, Stuart F. Quan, Elizabeth B. Klerman, Frank A. J. L. Scheer, Orfeu M. Buxton, Jonathan S. Williams, Charles A. Czeisler

**Affiliations:** ^1^Division of Sleep and Circadian Disorders, Departments of Medicine and Neurology, Brigham and Women’s Hospital, Boston, MA, United States; ^2^Division of Sleep Medicine, Harvard Medical School, Boston, MA, United States; ^3^Department of Biobehavioral Health, Pennsylvania State University, University Park, PA, United States; ^4^Division of Endocrinology, Diabetes, and Hypertension, Department of Medicine, Brigham and Women’s Hospital and Harvard Medical School, Boston, MA, United States

**Keywords:** chronic sleep restriction, recurrent circadian disruption, glucose tolerance, insulin sensitivity, metabolism

## Abstract

Insufficient sleep, which has been shown to adversely affect metabolism, is generally associated with prolonged exposure to artificial light at night, a known circadian disruptor. There is growing evidence suggesting that circadian disruption adversely affects metabolism, yet few studies have attempted to evaluate the adverse metabolic effects of insufficient sleep while controlling for circadian disruption. We assessed postprandial glucose and insulin responses to a standard breakfast meal in healthy adults (*n* = 9) who underwent 3 weeks of chronic sleep restriction (CSR) in a 37-day inpatient study while minimizing circadian disruption by maintaining the same duration of light exposure each study day. We compared these results to findings from an earlier inpatient study which used a forced desynchrony (FD) protocol to assess the influence of 3 weeks of CSR combined with recurrent circadian disruption (RCD) on glycemic control in healthy adults (*n* = 21). CSR combined with RCD resulted in significantly elevated postprandial plasma glucose levels (*p* < 0.0001), while CSR with minimized circadian disruption had no adverse glycemic effects after 3 weeks of exposure (EXP). These results suggest that one mechanism by which sleep restriction impacts metabolism may be via concurrent circadian disruption.

## Introduction

In a groundbreaking 1999 study, Spiegel and colleagues reported that 1 week of chronic sleep restriction (CSR) in healthy participants reduced glucose tolerance to a level associated with an increase in diabetes risk (Spiegel et al., 1999). Since then, numerous studies have found similar disturbances in glucose metabolism following varying amounts of sleep restriction (Nedeltcheva et al., 2009; Spiegel et al., 2009; Buxton et al., 2010; Donga et al., 2010; van Leeuwen et al., 2010; Schmid et al., 2011; Reynolds et al., 2012; Leproult et al., 2014; Eckel et al., 2015; Rao et al., 2015; Broussard et al., 2016; Wang et al., 2016; Depner et al., 2019; Ness et al., 2019a,b), selective suppression of slow-wave sleep (Tasali et al., 2008; Herzog et al., 2013) and untreated sleep-disordered breathing (Pallayova et al., 2010; Pamidi et al., 2015). It is now well established that sleep restriction decreases insulin sensitivity and glucose tolerance (Buxton et al., 2010; Ness et al., 2019a).

While multiple candidate causative mechanisms have been explored (Reutrakul and Van Cauter, 2018), the exact mechanisms by which sleep restriction elicits these effects is still unknown. In contrast to human studies, rodent studies have found that sleep deprivation or restriction can cause weight loss and improve glucose tolerance (Rechtschaffen et al., 1989; Everson and Crowley, 2004; Koban and Swinson, 2005; Caron and Stephenson, 2010; Vetrivelan et al., 2012). Importantly, most previous human studies of sleep restriction involved concurrent exposure to artificial light at night during extended wakefulness, which could have an adverse impact on glucose control through circadian disruption (Czeisler, 2013; Stenvers et al., 2019).

Previously, we reported that prior exposure to the combination of CSR and circadian disruption induced an increase in postprandial glucose levels associated with inadequate pancreatic insulin secretion, even when the circadian phase of participants was realigned so that there was no concurrent circadian misalignment (Buxton et al., 2012). To dissociate the two components of this exposure, in the present study we directly compared the influence of 3 weeks of CSR with recurrent circadian disruption (RCD) to 3 weeks of CSR while minimizing circadian disruption on glucose tolerance and insulin sensitivity in healthy adults.

## Materials and Methods

### Experimental Approach

**FIGURE 1:**
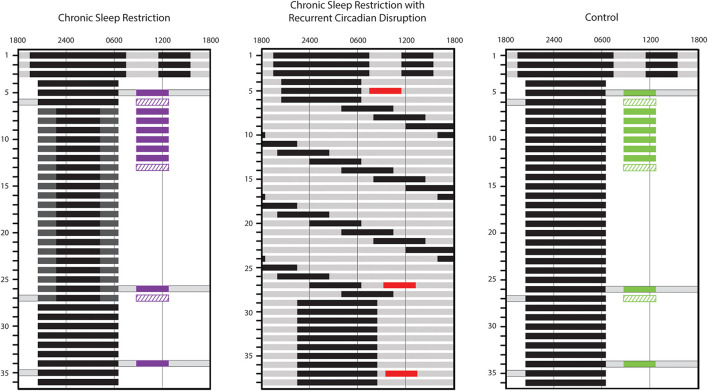
Inpatient study schedules. Study day is indicated along the left side and representative clock hour along the top. Solid black bars represent scheduled sleep episodes in 0 lux. Dark gray, light gray, and white indicate parts of the study conducted under 1 lux, 4 lux, and 90 lux lighting levels, respectively. Gray bars with black outlines indicate times during wake episodes where participants maintained a semi-recumbent posture in bed. The timing of standardized breakfast meal responses (solid colored bars; purple, red, and green used for chronic sleep restriction (CSR), CSR&RCD, and Control groups, respectively), for assessment of glucose and insulin and euglycemic-hyperinsulinemic clamps (diagonal hatched bars; purple and green used for CSR and Control groups, respectively) is also indicated. The standardized meal response on day 9 is available only in 4 CSR and 4 Control participants.

We report findings from three main study conditions: CSR (*n* = 9), Control (*n* = 8), and CSR&RCD (*n* = 21). All data were collected as part of a multi-study Program Project investigating the effects of sleep restriction and circadian disruption. Data from the CSR&RCD group were collected as part of a previous study (Buxton et al., 2012) and included here as a comparison to the CSR without circadian disruption group. Data from the CSR and Control groups were collected as part of the most recent study in the Program Project. Here, we focus on the effects of sleep restriction on glucose metabolism with and without circadian disruption (Figure 1).

The first four participants in the CSR group were studied under similar conditions to the CSR&RCD group, including a 10-h baseline (BL) sleep opportunity, sleep restriction to 5.6 h/24 h, and a lower-fat diet. For the remaining five CSR participants, we attempted to increase the metabolic challenge by reducing sleep opportunity to 5 h/24 h, introducing a higher-fat diet, and extending the eating window by reducing BL sleep to 8 h. Control participants were matched to the respective CSR BL conditions. After verifying that there were no significant differences in sleep duration at BL between the two CSR conditions or the two Control conditions, we combined the data to form a single CSR group (*n* = 9) and a single Control group (*n* = 8).

### Participant Recruitment and Eligibility Criteria

All participants were recruited from the community and screened to exclude medical, psychological, and sleep disorders. Participants did not take medication and had no acute or chronic illnesses. Screening included a medical history, physical examination, electrocardiogram, urinalysis, and clinical blood tests; psychological questionnaires (Minnesota Multiphasic Personality Inventory, Beck Depression Inventory) and a structured psychological interview with a clinical psychologist; and an overnight sleep recording [clinical polysomnography (PSG) or home sleep tests] to rule out clinically significant sleep disorders (Amira et al., 2020). Participants reported no significant sleep complaints, no history of regular night shift work for at least 3 years, and no travel across more than 2 time zones within 3 months before the study. Participants scheduled to all conditions had identical screening and selection criteria and were studied in the same research facility. Data collection procedures were standardized for all participants.

### Study Protocol

#### Pre-study Conditions

Participants in all conditions maintained a regular sleep-wake schedule for at least 3 weeks prior to admission, with 10-h per night time-in-bed at a self-selected time. Compliance was verified by wrist actigraphy (Actiwatch-S, Philips-Respironics, Murrysville, PA, United States or MotionWatch-8, CamNtech, Cambridge, United Kingdom), sleep diaries, and regular calls to a time-stamped phone answering system. During this time, participants were instructed not to use drugs, alcohol, nicotine, or caffeine. Compliance was verified with a urine toxicology screen upon study admission.

#### Inpatient Study Conditions

All studies took place in the Intensive Physiological Monitoring Unit of the Center for Clinical Investigation at Brigham and Women’s Hospital where participants were studied individually in private rooms free of time cues. The room was maintained at a temperature of 23.9 + 1.7°C, with complete darkness (<0.02 lux) during scheduled sleep opportunities. During wakefulness, participants could engage in sedentary activities such as reading, writing, etc. Research technicians observed participants throughout the study by closed circuit television to ensure compliance with the study protocol. All study events were timed relative to each participant’s habitual schedule.

In all conditions, the protocol began with three sleep extension days, each consisting of a 12-h nighttime sleep opportunity and a 4-h nap in the middle of the wake episode. These sleep extension days, in combination with the 3 weeks of regular sleep-wake schedule prior to admission, served to minimize the effect of any prior history of sleep loss. Sleep extension was followed by three BL days, each with a 10-h (CSR: *n* = 4; Control: *n* = 4; CSR&RCD: *n* = 21) (Buxton et al., 2012; McHill et al., 2018) or 8-h (CSR: *n* = 5; Control: *n* = 4) nighttime sleep opportunity. After BL, participants in the Control groups underwent an additional 3 weeks with the same BL sleep conditions. Room lighting was maintained at 90 lux during scheduled wake, except during sleep extension days and on three constant posture days when lighting was maintained at 4 lux and participants remained in bed in a semi-recumbent position (see Figure 1).

Participants in the CSR group with 10-h BL sleep opportunity then underwent 3 weeks of CSR with 5.6 h of sleep opportunity per night, centered around midsleep (with 2.2 h of extended wakefulness in near-darkness scheduled before and after the 5.6 h sleep opportunity). Participants in the CSR group with 8-h BL sleep opportunity underwent 3 weeks of CSR with 5 h of sleep opportunity per night, with the additional 3 h of extended wakefulness in near-darkness occurring before the sleep opportunity. To minimize circadian disruption, participants remained semi-recumbent in bed under ≤1 lux light levels during extended wakefulness (Figure 1). Thus, participants in the CSR group maintained the same 90-lux light exposure duration as during BL. Research technicians monitored the participants from within the room to ensure that participants remained awake while under 1 lux light levels.

In the CSR&RCD group, participants underwent 3 weeks of forced desynchrony (FD) consisting of 28-h “days,” each with a 21.47-h wake episode and a 6.53-h sleep opportunity (equivalent to 5.6 h/24 h of sleep). All wake episodes were scheduled in 4 lux lighting levels to avoid circadian phase-resetting properties of light.

Following the 3 weeks of exposure (EXP) to CSR&RCD, CSR, or Control conditions, all participants underwent 8 (CSR and Control groups) or 9 (CSR&RCD group) days of recovery (REC), each with the same nightly sleep opportunity they received during BL.

### Controlled Diet

During the inpatient study, participants received a controlled nutrient diet free of caffeine. In the CSR&RCD group and first four participants in each of the CSR and Control groups, this consisted of 55–60% carbohydrates, 15–20% protein, and 20–30% fat; in the subset of CSR and Control participants who received a higher-fat diet, carbohydrates were reduced to 30–40% to allow fat content to be increased to 45–50%. All diets also included 150 mEQ Na + (±20%), 100 mEq K + (±20%), and at least 1.5 liters/24 h of fluid. Breakfast meals were identical within participants during all meal response testing, and participants were required to finish all food. Kcals were adjusted when changes in weight exceeded 1 kg from average weight on inpatient days 3–4 to maintain stable participant weights throughout the study. Weighed foods confirmed that consumed kcals changed from BL by 2.63 ± 3.1% during the EXP segment of the protocols.

Calories were distributed evenly across breakfast, lunch, and dinner. In participants with the higher-fat diet, two additional snacks each consisting of 12.5% of the daily calories were added and the eating window was extended from 9.5 to 15 h. Meal timing was identical between the CSR and Control groups. The initial daily caloric intake was calculated using the Harris Benedict equation (activity factor 1.4) (Harris and Benedict, 1919) in the CSR&RCD group, and the Mifflin-St. Joer equation (Mifflin et al., 1990) for the first four participants in each of the CSR and Control groups (activity factor 1.3) and CSR and Control participants on the higher-fat diet (activity factor 1.6), both of which take into account the individual’s height, weight, age, and sex.

### Inpatient Study Measurements

#### Standardized Breakfast Meal Response

Standardized breakfast meal response tests for assessment of glucose and insulin were conducted during BL and the third week of EXP. In the CSR&RCD group, a day in the third week of EXP was chosen to ensure that the standardized meal occurred at the same circadian phase as the BL meal (60.8 ± 44.3 min). For participants in the Control and CSR groups, the meal response on day 26 was used as the 3-week EXP meal response, except for two disempaneled CSR participants who had the standardized meal on day 21, and one disempaneled Control participant who had the standardized meal on day 12. Participants in the Control and CSR groups on both diets also had multiple standardized breakfast meal responses conducted during the first week of EXP (Figure 1). The first half of participants (CSR: *n* = 4; Control: *n* = 4) had standardized meal responses every day of the first week, whereas the second half of participants (CSR: *n* = 5; Control: *n* = 4) are missing the meal response on study day 9. In each meal response, two fasted samples were collected via an indwelling catheter prior to breakfast. Blood samples were taken at minutes 10, 20, 30, 40, 50, 60, 90, 120, 150, 180, and 240 after the start of breakfast. Samples were placed on ice, centrifuged within 1 h of collection, aliquoted into separate tubes for glucose, insulin and lipids, and frozen at −80°C (glucose and insulin samples) or sent for assay within 48 h of collection (lipid samples).

#### Glucose and Insulin Assays and Analysis

Serum glucose was measured by Gluco-quant Glucose/HK kits (Roche Diagnostics GmbH, Mannheim, Germany) with a sensitivity of 2 mg/dL, an inter-assay precision CV of 1.7%, and an intra-assay precision CV of 1.0% (CSR&RCD group), or using the YSI 2300 STAT Plus Glucose and L-Lactate Analyzer (YSI Life Sciences) with a sensitivity of 2.5 mg/dL, an inter-assay precision CV of 2.3 to 6%, and an intra-assay precision CV of 1.4 to 1.8% (CSR and Control groups). Insulin was assayed using chemiluminescent immunoassay kits from Beckman Coulter, Inc. (Fullerton, CA, United States), with a sensitivity of 0.03 μIU/mL, an inter-assay precision of 3.1–5.6%, and an intra-assay precision of 2.0–4.2% (all groups). Postprandial glucose and insulin responses were quantified by calculating the Area-Under-the-Curve (AUC) from 0 through postprandial minute 180 with linear interpolation for missing values.

#### Insulin Sensitivity Assessments

Euglycemic-hyperinsulinemic clamps were performed in the first four participants in Control and CSR groups as previously described (Raji et al., 2001) during BL, week 1, week 3, and REC (Figure 1). We were unable to perform clamps in the remaining CSR and Control participants. Clamps were performed in the morning following an overnight fast, with participants remaining in bed for the procedure. Intravenous lines were placed in each arm for infusion or blood draw. After a BL sample was collected, participants were infused with human insulin (Novolin R) with priming doses of 80 and 60 mU/m^2^ body surface area per minute over the first two 5-min periods, respectively, followed by a constant infusion rate of 40 mU/m^2^ per minute for 170 min. Blood samples were collected every 5 min from *T* = 0 to *T* = 180 min from a catheter placed retrograde in a dorsal vein of the wrist; this hand was placed in a hand warmer thermostatically controlled at 60°C to arterialize venous blood. Serum glucose levels were determined immediately at the bedside. Dextrose solution (20%) was variably infused to maintain serum glucose levels at 90 mg/dL throughout the clamp procedure. To calculate the M-index, we calculated the mean dextrose infusion rate from at least 30 min of a steady-state (i.e., glucose levels consistently at 90 ± 5 mg/dL) from the last hour of the clamp (DeFronzo et al., 1979).

#### Lipid Assays and Analysis

Fasted serum samples were collected ∼60 min before the standardized breakfast during BL (days 5–7) and REC (days 34–36) and were assayed for triglycerides, total cholesterol and high-density lipoprotein (HDL); low-density lipoprotein (LDL) and very-low density lipoprotein (VLDL) were estimated using the Friedewald equation (Lipid Panel, #303756; LabCorp, Burlington, NC, United States). Values were calculated by averaging across samples within a study segment.

#### Body Weight and Body Fat Analysis

Wake-time, post-void weight was measured using a standard hospital scale and compared between BL, EXP, and REC days (Zitting et al., 2018). Pre- and post-study body composition was measured using Discovery W Dual-energy X-ray Absorptiometry (DXA) Scanner (Hologic, Bedford, MA, United States) in CSR and Control participants. Pre-study scans were performed ≤3 weeks before admission (except for one Control participant who was scanned ∼2 months before admission) and post-study scans were performed ≤2 days of discharge. Only a single scan ≤6 weeks of admission was performed for participants in the CSR&RCD group.

#### Polysomnographic Recording and Analysis

Ambulatory PSG was recorded throughout the study with a digital recorder (Vitaport 3, Temec Instruments, The Netherlands). The montage included electroencephalography (EEG; C3, C4, Fz, Cz, Pz, Oz referenced to linked mastoids M1 and M3), left and right electrooculography (EOGs), bipolar submental electromyography (EMG; during sleep episodes only), and bipolar electrocardiography (ECGs). Filtered and digitized EEG, EOG, EMG and ECG data were visually scored by a registered PSG technologist in 30 s epochs in Vitascore (Temec Instruments, The Netherlands) according to established criteria (33). Custom-built software (TASCI File Manager, developed at BWH by Mr. Joseph Ronda) was used to compile scored PSG data. Prorated average Total Sleep Time (TST) per 24 h was calculated for the last two BL sleep episodes, each of the EXP sleep episodes, and each of the REC sleep episodes.

#### Circadian Phase Assessments

For the CSR&RCD group, the intrinsic circadian period of the core body temperature data from the FD portion of the protocol was estimated using non-orthogonal spectral analysis (Czeisler et al., 1999). From this estimate, a circadian phase (from 0 to 359°) was assigned to each minute of the study, with 0°corresponding to the minimum of the waveform fit to the entire temperature data series. For participants in the CSR groups, 24-h melatonin profiles were assessed at BL, after ∼3 weeks of EXP, and at the end of REC. Dim Light Melatonin Onset (DLMO) was defined as the time at which plasma melatonin levels rose to 25% of the fitted nightly peak. Phase shifts were calculated as the difference in clock times of DLMO from BL to EXP or REC.

### Statistical Analysis

Statistical analyses were performed using SAS version 9.4 (SAS Institute, Cary, NC, United States). All outcomes were approximately normally distributed. *Post hoc* power analysis showed that we have 69% power to detect an effect size of 1.46 computed based on the 180 min AUC_*gluc*_ (two-sided two-sample *t*-test, *n* = 5, α = 0.05). Because the CSR and Control groups were part of a larger clinical trial with three total study arms, participants in the Control and CSR conditions were randomly assigned to one of three groups, stratified by gender and with a randomization block size of six. Linear mixed models were used to analyze the outcomes measured repeatedly over time from all groups whenever the data was available. Group, condition (BL, EXP, REC or Pre vs. Post for body fat), and group x condition were considered as fixed effects in the model, and subjects were considered as random effects. Gender, race (white, non-white), ethnicity (Hispanic, non-Hispanic), BMI, BL weight, and AHI were tested as covariates. Only significant covariates were retained in the final model. We included all available data from one participant in the Control group who was disempaneled on day 17. We were unable to obtain the REC meal response data for two CSR participants due to IV problems and a third who was disempaneled for fever on day 26. We do not have scored REC sleep data for four Control participants. Repeat participants were included as nested random effects. Imputation methods such as linear interpolation and last observation carried forward were only used when necessary for calculating AUCs, fasting glucose, and fasting lipids. All data are reported in text as mean ± SD, unless otherwise specified. The critical significance level was set at α = 0.05. The Bonferroni method was used to adjust for multiple comparisons and both unadjusted and Bonferroni-adjusted *p*-values are reported.

### Study Approval

The Partners Health Care Human Research Committee reviewed and approved this study (2005-P-002292; 2014-P-000243). The study conduct adhered to the ethical principles outlined in the Declaration of Helsinki and each participant provided written informed consent. This trial was registered on ClinicalTrials.gov (#NCT02171273).

## Results

### Participant Characteristics

A total of 18 studies in 15 participants were conducted under the Control and CSR experimental conditions. One CSR participant (57-year-old male) was disempaneled on study day 7 and not included in analyses. Thus, data are reported from 17 studies conducted in 14 participants: nine studies in the CSR group (67.1 ± 12.5 years, 27–71 years; 5 females); eight studies in the Control group (54.5 ± 8.5 years, 34–60 years, 3 females). Two CSR participants each completed the study twice under different diet conditions, and one participant completed the study twice under two different study arms (CSR and Control). No participant completed the study more than once under the same diet/experimental conditions. A total of 24 studies in 12 young and 12 older participants were conducted under CSR&RCD experimental conditions as reported previously (Buxton et al., 2012). One CSR&RCD participant (64-year-old male) was excluded due to IV access difficulties, and two CSR&RCD participants (58-year-old female and 19-year-old female) were excluded from analyses because blood samples were not obtained at the correct circadian phase. Therefore, data are reported from 21 participants in the CSR&RCD group (40.1 ± 19.1 years, 18–70 years; 10 females). For participant characteristics, see Supplementary Table 1.

### Sleep and Circadian Phase Assessments

As expected, there was a significant reduction in TST during EXP compared to BL in the CSR group (*n* = 9; LSMdiff: 138 min; bootstrapping 95% CI (102, 172), unadjusted empirical bootstrap *p* < 0.0001, *p*_*bon*_ < 0.0001) and the CSR&RCD group (*n* = 21; LSMdiff: 170 min; bootstrapping 95% CI (135, 201), unadjusted empirical bootstrap *p* < 0.0001, *p*_*bon*_ < 0.0001). In the CSR group, TST did not recover to BL levels (LSMdiff: 80 min; bootstrapping 95% CI (25, 143), unadjusted empirical bootstrap *p* = 0.003, *p*_*bon*_ = 0.018). In the CSR&RCD group, there was no significant difference in TST between BL and REC. We also found no significant difference in TST among BL, EXP, and REC time points in the Control (*n* = 8) group (Supplementary Table 2).

To confirm that we successfully minimized circadian disruption in the CSR group, we assessed DLMO at BL, EXP, and REC and found no significant circadian phase shifts at any time points (Supplementary Table 2).

### Postprandial Glucose Levels After 3 Weeks of Chronic Sleep Restriction While Minimizing Circadian Disruption or Chronic Sleep Restriction Combined With Recurrent Circadian Disruption

**FIGURE 2:**
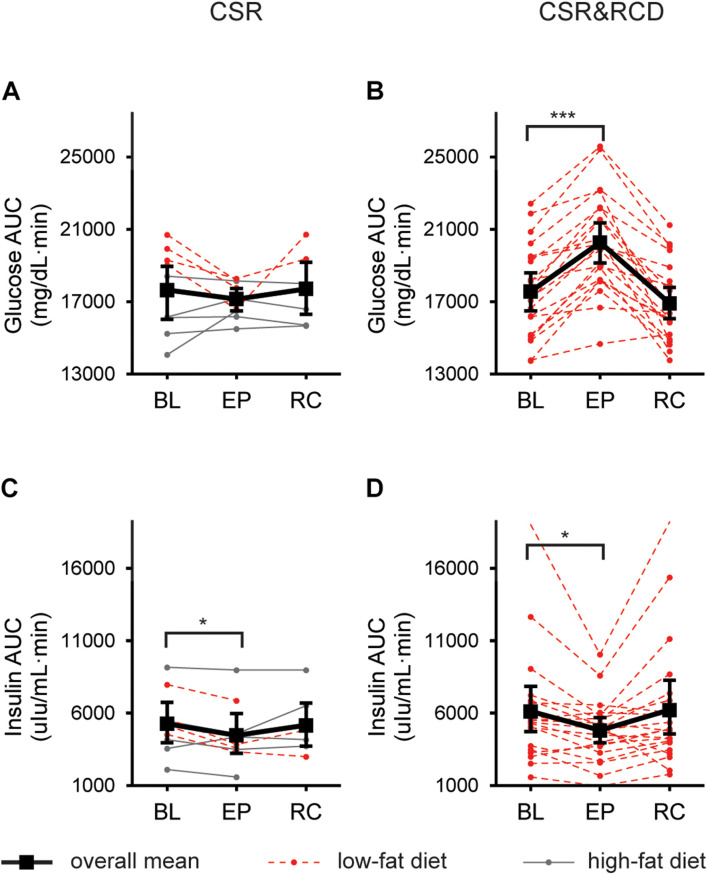
Postprandial glucose and insulin responses to a standardized breakfast meal after 3 weeks of CSR with and without concurrent recurrent circadian disruption (RCD). Glucose and insulin 180-min Area-Under-the-Curves (AUCs) at baseline (BL), after 3 weeks of exposure (EXP), and near the end of recovery (REC) in the CSR group (*n* = 9; **A**,**C**), and the CSR&RCD group (*n* = 21; **B**,**D**). For postprandial glucose and insulin responses in the Control groups, see Zitting and Vetrivelan (submitted). LSmeans and 95% bootstrapping confidence intervals are shown in black, while individual raw data is shown in dotted red lines (low-fat diet) or solid gray lines (high-fat diet). Bonferroni-adjusted bootstrapping *p*-values are indicated as follows: *p ≤ 0.05 and ***p ≤ 0.001.

In the CSR group (*n* = 9), in which circadian disruption was minimized, there was no significant change in AUC_*gluc*_ at EXP or REC compared to BL (Figure 2A). In contrast, in the CSR&RCD group (*n* = 21), there was a significant increase in AUC_*gluc*_ at EXP when compared to BL (LSMdiff: 2726; bootstrapping 95% CI (2105, 3293), unadjusted empirical bootstrap *p* < 0.0001, *p*_*bon*_ < 0.0001). AUC_*gluc*_ was not different at REC compared to BL in the CSR&RCD group (Figure 2B). There were no significant differences in AUC_*gluc*_ in the Control group (*n* = 8) at BL, EXP, and REC (Zitting and Vetrivelan, submitted).

In a subset of CSR participants (*n* = 4) who were on a low-fat diet, we observed a significant overall decrease in AUC_*gluc*_ at EXP compared to BL (LSMdiff: 2033; bootstrapping 95% CI (1359, 2487), unadjusted empirical bootstrap *p* < 0.0001, *p*_*bon*_ < 0.0001) which was not different at REC compared to BL. The remaining CSR participants (*n* = 5) showed no significant differences in AUC_*gluc*_ at any point.

### Postprandial Insulin Levels After 3 Weeks of Chronic Sleep Restriction While Minimizing Circadian Disruption or Chronic Sleep Restriction Combined With Recurrent Circadian Disruption

In the CSR group, there was a decrease in AUC_*ins*_ when comparing BL to EXP (LSMdiff: 790; bootstrapping 95% CI (267, 1228), unadjusted empirical bootstrap *p* = 0.009, *p*_*bon*_ = 0.054). AUC_*ins*_ was not significantly different at REC compared to BL (Figure 2C). In the CSR&RCD group, there was a decrease in AUC_*ins*_ at EXP compared to BL (LSMdiff: 1317; bootstrapping 95% CI (555, 2331), unadjusted empirical bootstrap *p* = 0.015, *p*_*bon*_ = 0.09). AUC_*ins*_ was not different at REC compared to BL in the CSR&RCD group (Figure 2D). There were no significant changes in AUC_*ins*_ in the Control group (Zitting and Vetrivelan et al., submitted).

In a subset of CSR participants (*n* = 4) who were on a low-fat diet, we found a significant decrease in AUC_*ins*_ at EXP compared to BL (LSMdiff: 1405; bootstrapping 95% CI (1148, 1719), unadjusted empirical bootstrap *p* < 0.0001, *p*_*bon*_ < 0.0001), which then remained low at REC compared to BL (LSMdiff: 1182; bootstrapping 95% CI (686, 1728), unadjusted empirical bootstrap *p* < 0.0001, *p*_*bon*_ < 0.0001). The remaining CSR participants (*n* = 5) showed no significant differences in AUC_*ins*_ at any point.

### Fasting Glucose Levels After 3 Weeks of Chronic Sleep Restriction While Minimizing Circadian Disruption or Chronic Sleep Restriction Combined With Recurrent Circadian Disruption

There was no change in fasting glucose levels after 3 weeks of CSR while minimizing circadian disruption between BL, EXP, and REC. In contrast, in the CSR&RCD group, there was a significant increase in fasting glucose levels at EXP compared to BL (LSMdiff: 5.41; bootstrapping 95% CI (3.12, 7.97), unadjusted empirical bootstrap *p* < 0.0001, *p*_*bon*_ < 0.0001), which then recovered to below BL levels (LSMdiff: 3.02; bootstrapping 95% CI (0.66, 5.66), unadjusted empirical bootstrap *p* = 0.014, *p*_*bon*_ = 0.084). There were no changes in fasting glucose levels between BL, EXP, and REC in the Control group (Zitting and Vetrivelan et al., submitted).

### Daily Assessments of Postprandial Glucose and Insulin Levels During the First Week of Chronic Sleep Restriction While Minimizing Circadian Disruption

**FIGURE 3:**
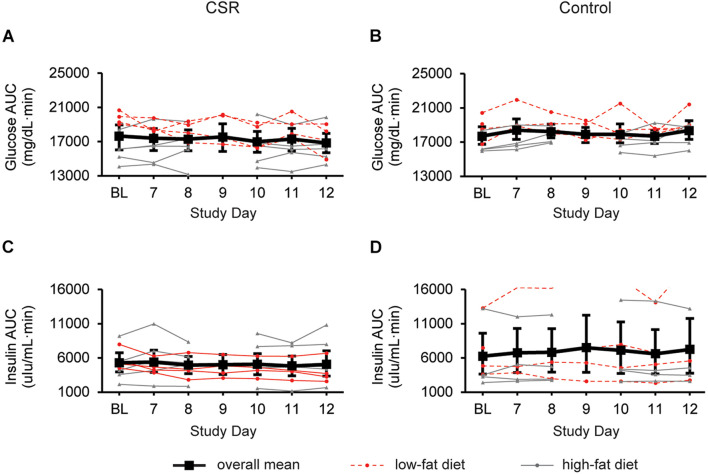
Glucose and insulin responses to a standardized breakfast meal at BL and during week 1 of CSR without circadian disruption or Control. 180-min glucose and insulin AUCs for CSR (*n* = 9; **A**,**C**) and Control (*n* = 8; **B**,**D**) groups. LSmeans and 95% bootstrapping confidence intervals are shown in black, while individual raw data is shown in dotted red lines (low-fat diet) or solid gray lines (high-fat diet).

We found no significant change in AUC_*gluc*_ (Figure 3A) or AUC_*ins*_ (Figure 3C) AUC during the first week of EXP compared to BL in the CSR group. Unexpectedly, in a subset of participants (*n* = 4) in the CSR group who were on a low-fat diet, we found that AUC_*gluc*_ in the first week of EXP was significantly lower than BL (LSMdiff: 1508; bootstrapping 95% CI (840, 2030), unadjusted empirical bootstrap *p* < 0.0001, *p*_*bon*_ < 0.0001; Supplementary Figure 1A). Similarly, AUC_*in*__*s*_ was lower during the first week of EXP compared to BL in this group only (LSMdiff: 1381; bootstrapping 95% CI (941, 1663), unadjusted empirical bootstrap *p* < 0.0001, *p*_*bon*_ < 0.0001; Supplementary Figure 1B). In the remaining CSR participants who were on the higher-fat diet, we observed no significant change in either AUC_*gluc*_ or AUC_*ins*_.

### Daily Assessments of Postprandial Glucose and Insulin Levels During the First Week Under Control Conditions

In the Control group, there were no significant changes in AUC_*gl*__*uc*_ (Figure 3B) or AUC_*ins*_ (Figure 3D) during the first week in the laboratory.

### Insulin Sensitivity During 3 Weeks of Chronic Sleep Restriction While Minimizing Circadian Disruption

**FIGURE 4:**
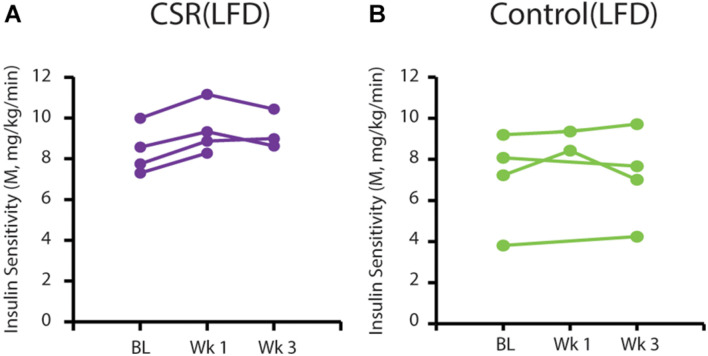
Insulin sensitivity on a low-fat diet at BL and after 3 EXP weeks of CSR or Control conditions. M-index calculated from euglycemic-hyperinsulinemic clamps conducted at BL, after 1 week of CSR or Control EXP conditions (Wk 1), and after 3 EXP weeks of CSR or Control conditions (Wk 3). Each line represents data from an individual participant in the **(A)** CSR group (purple; *n* = 4) or **(B)** Control group (green; *n* = 4).

We performed euglycemic-hyperinsulinemic clamps in the first half of CSR (*n* = 4) and Control (*n* = 4) participants during BL, and after the first and third weeks of EXP. We detected no overall effects of condition in the CSR or Control group on insulin sensitivity (Figure 4).

### Body Weight, Body Fat, and Fasting Lipids

Chronic sleep restriction participants showed no significant change in weight throughout the study. In the CSR&RCD group, participants exhibited a significant but modest reduction in mean body weight at EXP compared to BL (LSMdiff: 0.97 kg; bootstrapping 95% CI (0.59, 1.34), unadjusted empirical bootstrap *p* < 0.0001, *p*_*bon*_ < 0.0001) and continued to lose weight during REC, resulting in a small but significant reduction in body weight (LSMdiff: 1.22 kg; bootstrapping 95% CI (0.74, 1.66), unadjusted empirical bootstrap *p* < 0.0001, *p*_*bon*_ < 0.0001). There was no significant change in weight in the Control participants. Dual-energy X-ray absorptiometry scans performed before and after the study revealed no significant changes in total body fat within any of the groups (Supplementary Table 2). No significant changes were observed in the levels of any fasting lipids in the CSR, Control, and CSR&RCD groups (Supplementary Table 2).

## Discussion

We previously reported that 3 weeks of CSR with RCD increased glucose levels in response to a standardized breakfast meal due to inadequate insulin levels (Buxton et al., 2012). In the present study, we found that glucose levels were not increased in response to a standardized breakfast meal after 3 weeks of CSR while minimizing circadian disruption. Notably, we have also found in the same individuals that slow wave activity and slow wave sleep duration increased during 3 weeks of CSR (Xin and Yuan et al., submitted). Given the important role of slow wave sleep in glucose metabolism (Tasali et al., 2008; Herzog et al., 2013), its preservation in the CSR condition when minimizing circadian disruption may account for why glucose metabolism was not impaired.

In a subset of CSR participants who were on a low-fat diet, near-daily assessments of glucose and insulin metabolism during week 1 of EXP to CSR revealed a significant decrease in postprandial glucose and insulin levels in response to the standardized test meal, suggesting improved glucose tolerance and insulin sensitivity. Although unexpected, this result is consistent with our overall finding that exposure to CSR while minimizing circadian disruption has no adverse effect on glucose tolerance. Unlike real-world sleep restriction, these participants were not permitted to consume additional calories nor were they exposed to light at night during their extended wake. Therefore, the observed improvement in glucose tolerance in these participants could potentially be due to increased caloric utilization as a result of their extended wakefulness; the combination of CSR with a low-fat diet would have inadvertently resulted in greater caloric restriction compared to the other CSR participants who were on a more typical western diet. Overall, these findings—that CSR while minimizing circadian disruption has a minimal adverse impact on glucose metabolism—are similar to a recent study of chronic partial sleep loss in rats showing slower weight gain, decreased plasma glucose, and no changes in plasma insulin levels after 60 days of exposure to sleep deficiency while on a low-fat diet (16.7% fat) of regular rat chow (Vetrivelan et al., 2012).

Many human studies have demonstrated impaired glucose regulation and/or insulin sensitivity after as few as 1–7 days of sleep deprivation or CSR (Nedeltcheva et al., 2009; Buxton et al., 2010; Donga et al., 2010; van Leeuwen et al., 2010; Schmid et al., 2011; Reynolds et al., 2012; Leproult et al., 2014; Rao et al., 2015; Broussard et al., 2016; Wang et al., 2016; Ness et al., 2019a). However, in those studies, sleep restriction was also associated with circadian disruption through extension of the daily photoperiod, i.e., the duration of light exposure in a 24-h day. Circadian misalignment has itself been shown to disrupt glucose metabolism, with an important role for impaired insulin sensitivity and muscle non-oxidative glucose disposal (Scheer et al., 2009; Leproult et al., 2014; Qian et al., 2018; Wefers et al., 2018). Moreover, changes in photoperiod have been shown to impact metabolism in a wide array of species (Rousseau et al., 2003; Walton et al., 2011). In the present study, we maintained the same photoperiod in the EXP segment as in the BL by keeping participants in near darkness during extended wakefulness, thereby minimizing the confounding effects of extended exposure to light. Furthermore, we performed our primary assessments after 3 weeks of EXP, allowing time for adaptation. It may be that the extended photoperiod inherent in prior CSR studies contributed to the reported metabolic effects by affecting melatonin concentrations –which may be a key regulator of glucose metabolism (McMullan et al., 2013a,b; Rubio-Sastre et al., 2014; Garaulet et al., 2015, 2020; Lopez-Minguez et al., 2018; Karamitri and Jockers, 2019) –either by delaying the evening rise in melatonin or by reducing the duration of the diurnal profile of melatonin. Of note, Leproult et al. (2014) found that recent exposure to extreme circadian misalignment coupled with CSR had significantly larger adverse effects on key diabetes risk factors than CSR alone. Consistent with these findings, our results support the hypothesis that circadian disruption may contribute to the increased diabetes risk that has been associated with chronic insufficient sleep (Committee on Sleep Medicine and Research, Board on Health Sciences Policy, 2006).

*Limitations*: We compared the effects of 3 weeks of CSR in highly controlled inpatient studies under two conditions: (1) while minimizing circadian disruption, and (2) while imposing RCD. As the participants in condition 2 were from a previous study, we could not randomize participants between the two groups or do direct statistical comparisons between groups. However, given the hundreds of published studies documenting the association between sleep restriction and impaired glucose metabolism, our findings are both unexpected and noteworthy that in carefully controlled laboratory conditions, nine healthy individuals studied for 3 weeks of EXP to CSR with minimal circadian disruption showed no impairment of glucose metabolism. These studies, which involved 1,152 days of hospitalization and represent some of the longest inpatient studies of CSR, break open a new field of inquiry regarding the role of circadian disruption in the pathophysiology of sleep restriction-induced impairment of glucose metabolism in humans.

## Conclusion

In summary, we found that while 3 weeks of CSR combined with RCD impaired glucose tolerance, 3 weeks of CSR while minimizing circadian disruption had no adverse effect on glucose metabolism. These results suggest that the mechanism by which chronic sleep loss impacts metabolic health may require circadian disruption and/or fragmentation [e.g., sleep apnea (Pamidi et al., 2015)] or selective deprivation of slow wave sleep (Tasali et al., 2008). Future studies are needed to clarify how exposure to extended duration artificial light at night, melatonin secretion, meal timing, and diet interact with sleep loss to impact metabolism.

## Data Availability Statement

The datasets presented in this article are not readily available because execution of a materials transfer agreement is required by our institution for transfer of data. Requests to access the datasets should be directed to CC, cacadmin@partners.org.

## Ethics Statement

The studies involving human participants were reviewed and approved by the Partners Health Care Human Research Committee. The patients/participants provided their written informed consent to participate in this study.

## Author Contributions

CC, OB, JD, and JW designed the experiments. RY, K-MZ, and NV conducted the experiments. RY, K-MZ, NV and WW analyzed the data. RY and K-MZ prepared the figures and tables. RY, K-MZ, NV, JW, JD, OB, EK, SQ, FS, and CC wrote the manuscript. All authors reviewed the manuscript. RY and K-MZ were the project leaders for the experiments.

## Conflict of Interest

EK has received travel support from the Society of Reproductive Investigation, the Sleep Research Society, the National Sleep Foundation, the World Conference of Chronobiology, the Gordon Research Conferences, the Santa Fe Institute, and the DGSM; and consulting fees from Pfizer Inc., the Puerto Rico Trust, the National Sleep Foundation, Sanofi-Genzyme, and Circadian Therapeutics. FS has received lecture fees from Bayer HealthCare, Sentara HealthCare, Philips, Vanda Pharmaceuticals, and Pfizer Pharmaceuticals. SQ has received consulting fees from Jazz Pharmaceuticals and Best Doctors, and is a consultant to Whispersom. OB has received subcontracts to Penn State from Mobile Sleep Technologies/Proactive Life (National Science Foundation #1622766, National Institutes of Health R43AG056250, R44 AG056250), honoraria/travel support for lectures from Boston University, Boston College, Tufts School of Dental Medicine, Allstate, consulting fees from SleepNumber, and receives an honorarium for his role as the Editor in Chief (designate) of Sleep Health sleephealthjournal.org. CC reports grants from Cephalon Inc., Jazz Pharmaceuticals Plc., Inc., National Football League Charities, Optum, Philips Respironics, Inc., Regeneron Pharmaceuticals, ResMed Foundation, San Francisco Bar Pilots, Sanofi S.A., Sanofi-Aventis, Inc., Schneider Inc., Sepracor, Inc., Mary Ann and Stanley Snider via Combined Jewish Philanthropies, Sysco, Takeda Pharmaceuticals, Teva Pharmaceuticals Industries, Ltd., and Wake Up Narcolepsy; and personal fees from Bose Corporation, Boston Celtics, Boston Red Sox, Cephalon, Inc., Columbia River Bar Pilots, Ganésco Inc., Institute of Digital Media and Child Development, Klarman Family Foundation, Samsung Electronics, Quest Diagnostics, Inc., Teva Pharma Australia, Vanda Pharmaceuticals, Washington State Board of Pilotage Commissioners, Zurich Insurance Company, Ltd. In addition, CC holds a number of process patents in the field of sleep/circadian rhythms (e.g., photic resetting of the human circadian pacemaker) and holds an equity interest in Vanda Pharmaceuticals, Inc. Since 1985, CC has also served as an expert on various legal and technical cases related to sleep and/or circadian rhythms, including those involving the following commercial entities: Casper Sleep Inc., Comair/Delta Airlines, Complete General Construction Company, FedEx, Greyhound, HG Energy LLC, Purdue Pharma, LP, South Carolina Central Railroad Co., Steel Warehouse Inc., Stric-Lan Companies LLC, Texas Premier Resource LLC, and United Parcel Service (UPS). CC receives royalties from the New England Journal of Medicine; McGraw Hill; Houghton Mifflin Harcourt/Penguin; and Philips Respironics, Inc., for the Actiwatch-2 and Actiwatch-Spectrum devices. CC’s interests were reviewed and managed by Brigham and Women’s Hospital and Partners HealthCare in accordance with their conflict of interest policies. The remaining authors declare that the research was conducted in the absence of any commercial or financial relationships that could be construed as a potential conflict of interest.

## Publisher’s Note

All claims expressed in this article are solely those of the authors and do not necessarily represent those of their affiliated organizations, or those of the publisher, the editors and the reviewers. Any product that may be evaluated in this article, or claim that may be made by its manufacturer, is not guaranteed or endorsed by the publisher.
