# Supplementary material for: Chronic Sleep Restriction While Minimizing Circadian Disruption Does Not Adversely Affect Glucose Tolerance
Source: Front Physiol. 2021 Oct 20;12:764737. doi: 10.3389/fphys.2021.764737 (PMC8564292; doi:10.3389/fphys.2021.764737)
Supplement: Supplementary file 1 [file Data_Sheet_1.docx]

Supplementary Material

# Supplementary Figures and Tables

## Supplementary Figures


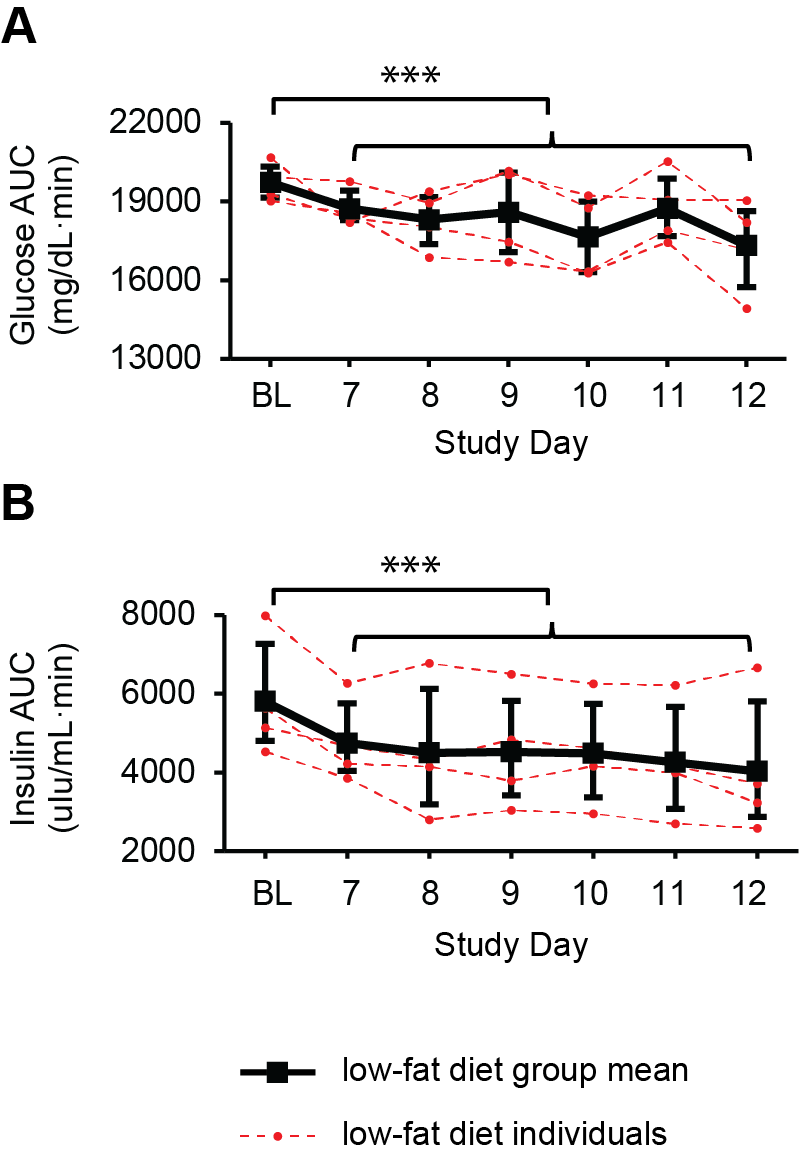


**Supplementary Figure 1.** **Glucose and insulin responses to a standardized breakfast meal at baseline and during week 1 of CSR on a low-fat diet.** 180-minute glucose (A) and insulin (B) AUCs for the first half of CSR participants who were on a low-fat diet (n=4). LSmeans and 95% bootstrapping confidence intervals are shown in black, while individual raw data is shown in dotted red lines. ***p<0.0001.

## Supplementary Tables

|  |  | LFD | LFD | LFD | HFD | HFD |
| --- | --- | --- | --- | --- | --- | --- |
|  |  | CSR&RCD | CSR | Control | CSR | Control |
|  |  | (*N* = 21) | (*N* = 4) | (*N* = 4) | (*N* = 5) | (*N* = 4) |
|  |  | Mean (range) | | | | |
|  | Age (years) | 40.1 (18-70) | 62.8 (56-71) | 57.0 (55-60) | 52.6 (27-65) | 52.0 (34-60) |
|  | Sex |  |  |  |  |  |
|  | Men, n (%) | 11 (52.4) | 1 (25.0) | 2 (50.0) | 3 (60.0) | 3 (75.0) |
|  | Women, n (%) | 10 (47.6) | 3 (75.0) | 2 (50.0) | 2 (40.0) | 1 (25.0) |
|  | Race/Ethnicity |  |  |  |  |  |
|  | Caucasian/Hispanic,  n (%) | 2 (9.5) | 0 (0.0) | 1 (25.0) | 1 (20.0) | 2 (50.0) |
|  | Other/Hispanic, n (%) | 1 (4.8) | 0 (0.0) | 0 (0.0) | 0 (0.0) | 0 (0.0) |
|  | Caucasian/Non-Hispanic, n (%) | 16 (76.2) | 3 (75.0) | 3 (75.0) | 2 (40.0) | 2 (50.0) |
|  | African-American,  n (%) | 0 (0.0) | 0 (0.0) | 0 (0.0) | 1 (20.0) | 0 (0.0) |
|  | Asian, n (%) | 0 (0.0) | 1 (25.0) | 0 (0.0) | 0 (0.0) | 0 (0.0) |
|  | More than one, n (%) | 2 (9.5) | 0 (0.0) | 0 (0.0) | 1 (20.0) | 0 (0.0) |
|  | Sleep Quality |  |  |  |  |  |
|  | PSQI score | N/A | 3.0 (1-6) | 1.8 (1-3) | 1.8 (1-4) | 2.8 (0-4) |
|  | ESS | N/A | 4.5 (1-7) | 3.8 (1-9) | 3.6 (0-7) | 3.8 (1-6) |
|  | AHI | N/A | 3.9 (2.5-6.9) | 4.0 (1.0-6.6) | 5.4 (2.6-13.7) | 6.6 (1.3-13.7) |
|  | BMI (kg/m2) | 23.9 (18.8-29.4) | 21.5 (19.4-23.3) | 25.6 (19.9-29.9) | 25.7 (20.0-29.9) | 25.2 (21.1-29.6) |
|  |  |  |  |  |  |  |

Table S1. Characteristics of participants included in analysis.

Abbreviations: Lower-fat diet (LFD), High-fat diet (HFD), Chronic Sleep Restriction with Recurrent Circadian Disruption (CSR&RCD), Chronic Sleep Restriction (CSR). Prior to admission, participants were assessed on the Pittsburgh Sleep Quality Index (PSQI), Epworth Sleepiness Scale (ESS), Apnea-Hypopnea Index (AHI), and Body Mass Index (BMI).

|  |  |  | CSR&RCD | CSR | Control |  |
| --- | --- | --- | --- | --- | --- | --- |
|  |  |  | (*N* = 21) | (*N* = 9) | (*N* = 8) |  |
|  |  |  | Mean (range) | | |  |
|  | Total sleep time  (TST, hours) | BL | 7.5 (4.5-9.1) | 6.9 (4.6-8.1) | 6.3 (4.8-8.6) |  |
|  |  | EXP | 5.1 (3.8-5.5)* | 4.5 (3.4-5.2)* | 6.8 (5.2-8.1) |  |
|  |  | REC | 8.0 (5.3-9.2) | 6.1 (4.0-7.6)^A^* | N/A |  |
|  | DLMO shift  (hours) | BL vs. EXP | N/A | -0.6 (-1.7-0.9)^B^ | N/A |  |
|  |  | BL vs. REC | N/A | -0.6 (-1.5-0.2)^B^ | -0.4 (-1.5-0.3)^C^ |  |
|  | Total body fat  (% of body mass) | PRE | 27.6 (10.6-40.3) | 33.4 (27.0-47.5) | 34.8 (20.0-47.0)^E^ |  |
|  |  | POST | N/A | 33.2 (28.6-47.6)^D^ | 35.0 (21.5-44.7)^F^ |  |
|  | Triglycerides | BL | 108.4 (42.0-217.0)^G^ | 86.4 (41.7-134.0) | 112.0 (77.0-146.5) |  |
|  | Total cholesterol |  | 178.6 (107.0-277.0)^G^ | 183.9 (154.0-218.3) | 183.8 (161.5-231.5) |  |
|  | HDL cholesterol |  | 44.8 (29.0-65.0)^G^ | 55.4 (42.0-95.5) | 46.2 (33.0-55.0) |  |
|  | LDL cholesterol |  | 112.1 (60.0-191.0)^G^ | 111.1 (87.5-148.0) | 115.3 (91.0-157.0) |  |
|  | VLDL cholesterol |  | 21.7 (8.0-43.0)^G^ | 17.3 (8.3-26.7) | 22.3 (15.0-29.0) |  |
|  | Triglycerides | REC | 105.7 (44.0-210.0) | 82.3 (51.0-155.7)^H^ | 104.8 (80.5-132.0)^I^ |  |
|  | Total cholesterol |  | 168.1 (108.0-244.0) | 178.9 (154.5-219.7)^H^ | 172.2 (138.0-216.0)^I^ |  |
|  | HDL cholesterol |  | 44.5 (28.0-63.0) | 54.8 (39.7-83.5)^H^ | 44.8 (30.0-55.0)^I^ |  |
|  | LDL cholesterol |  | 102.8 (62.0-162.0) | 107.7 (78.5-149.0)^H^ | 106.4 (88.0-140.0)^I^ |  |
|  | VLDL cholesterol |  | 20.8 (9.0-42.0) | 16.4 (10.0-31.0)^H^ | 21.0 (16.0-26.5)^I^ |  |

Table S2. Participant sleep, DLMO, body fat, and lipid levels.

Abbreviations: High-density lipoprotein (HDL), low-density lipoprotein (LDL), very low-density lipoprotein (VLDL), dim light melatonin onset (DLMO), baseline (BL), exposure (EXP), recovery (REC).

^A^ REC sleep was missed for two CSR participants

^B^ BL vs. EXP DLMOs were missed for two CSR participants and BL vs. REC DLMOs were missed for three CSR participants.

^C^ BL vs. EXP and BL vs. REC DLMO were missed for two CTRL participants.

^D^ Post-DXA scan was missed for three CSR participants

^E^ Pre-DXA scan was missed for one Control participant

^F^ Post-DXA scan was missed for one Control participant

^G^ Baseline fasting lipids were missed for two CSR&RCD participants

^H^ REC fasting lipids were missed for three CSR participants.

^I^ REC fasting lipids were missed for one Control participant.

*p<0.05 for comparisons against BL

Note that in the CSR&RCD group, only a single DXA scan was obtained from each participant.
